# Choroidal vascular changes in silicone oil-filled eyes after vitrectomy for rhegmatogenous retinal detachments

**DOI:** 10.1186/s12886-023-03167-x

**Published:** 2023-11-02

**Authors:** Jiayu Chen, Lina Guan, Yalu Liu, Yingying Song, Yu Tang, Yumei Cao, Meishuang Li, Aiqin Sheng, Zhengpei Zhang, Haiyang Liu

**Affiliations:** 1 The Affiliated Xuzhou Municipal Hospital of Xuzhou Medical University, 269 Daxue Road, Tongshan District, Xuzhou, 221000 China; 2 Department of Ophthalmology, Xuzhou First People’s Hospital, 269 Daxue Road, Tongshan District, Xuzhou, 221000 China; 3 Xuzhou Medical University, 209 Tongshan Road, Yunlong District, Xuzhou, 221004 China; 4 Eye Disease Prevention and Treatment Institute of Xuzhou, 269 Daxue Road, Tongshan District, Xuzhou, 2210004 China

**Keywords:** Rhegmatogenous retinal detachment, Silicone oil tamponade, Choroidal vascular index, Choroidal thickness, Optical coherence tomography

## Abstract

**Introduction:**

The tamponade of silicone oil (SO) can affect both the structure and blood flow of the retina. However, there are few studies on the effect of SO tamponade on choroidal blood flow. Our study aimed to compare the effects of SO tamponade on the choroidal vascular index (CVI) and choroidal thickness (CT) in patients with unilateral rhegmatogenous retinal detachment (RRD) with operated eyes and fellow healthy eyes.

**Methods:**

We retrospectively collected demographic and clinical data from 36 patients who underwent 23G pars plana vitrectomy and SO tamponade for unilateral complicated RRD. Enhanced depth imaging-optical coherence tomography (EDI-OCT) scans were performed both within 1 week before SO removal and at the last follow-up visit after SO removal. Using ImageJ software, images were binarized to segment the total choroidal area, luminal area, and stromal area, respectively. The CVI was calculated as CVI=(luminal area)/(total choroidal area), and CT was also evaluated.

**Results:**

During SO tamponade, the CVI and luminal area in operated eyes were significantly lower compared to fellow eyes (57.616 ± 0.030 vs. 60.042 ± 0.019, P < 0.0001; 0.909 [0.694; 1.185] vs. 1.091 [0.785; 1.296], P = 0.007). Even after SO removal, the CVI remained lower in operated eyes than in fellow eyes (59.530 ± 0.018 vs. 60.319 ± 0.020, P = 0.031). Both CVI and luminal area were lower in operated eyes before SO removal than after SO removal (57.616 ± 0.030 vs. 59.530 ± 0.018, P = 0.0003; 0.909 [0.694; 1.185] vs. 0.994 [0.712; 1.348], P = 0.028). The duration of SO tamponade was positively correlated with the difference in CVI between fellow eyes and operated eyes during SO tamponade (P = 0.035). Total choroidal area, stromal area, and CT did not differ significantly between fellow eyes and operated eyes or between pre- and post-SO removal.

**Conclusions:**

SO tamponade reduces CVI and decreases choroidal blood circulation in patients with retinal detachments required vitrectomy combined with SO tamponade. The longer the SO tamponade time, the more CVI reduction. In future work, we will aim to reduce these side effects by shortening the duration of silicone oil filling.

## Introduction

Retinal detachment is a disease that seriously affects visual function and is currently treated mainly by surgery, mainly including scleral buckling and vitrectomy. Vitrectomy requires intraocular tamponade, including air, expanding gas (e.g., C_3_F_8_), and silicone oil [1]. Some patients with monocular retinal detachment are not detected in time and come to the hospital with a more serious symptoms, and silicone oil is necessary for intraocular tamponade. Silicone oil cannot be kept in the eye for a long period of time, and after the good reset of the retina, it needs to be removed from the eye. The retention time of silicone oil is inconclusive, generally in the range of 3–6 months [2, 3]. It is necessary to conduct a study on whether silicone oil has an impact on the retina, choroid, and related structures after repositioning, as well as whether there is a correlation between the retention time of silicone oil and this effect.

Numerous studies have revealed a significant reduction in central macular thickness (CMT) during SO tamponade, which subsequently recovers after SO removal. The thinning of CMT during SO tamponade is primarily attributed to the inner retinal layer’s thinning [4, 5]. The CMT reduction was considerably more pronounced in the SO-filled group than in the normal contralateral group [6]. Additionally, SO tamponade has been found to affect not only the thickness of the retina but also the retina’s blood flow, as determined by Fluorescein angiography and Optical Coherence Tomography Angiography. Compared to the contralateral eye, the SO-filled group exhibited a decrease in retinal superficial capillary flow density, deep capillary flow density, and total retinal flow density [7–9]. Meanwhile, the retention time of silicone oil is significantly correlated with the expansion of the deep capillary layer of the foveal avascular zone (FAZ) and the decrease in blood flow density [6].

The choroid is considered to be the sole source of blood supply to the FAZ of the retina [10–12]. In the past, choroidal thickness (CT) was used as an indicator of choroidal structure. However, studies have shown that when SO is filled for more than 6 months, there is significant thinning of the CT under the central macular foveal, and no improvement in CT is observed after removal of SO. Additionally, when SO is filled for more than 9 months, the thinning of CT is correlated with decreased best corrected visual acuity (BCVA) [13–16]. Subfoveal CT thinning can cause outer retinal dysfunction and negatively affect the recovery of visual function [17]. Although some studies suggest that silicone oil tamponade has no significant effect on choroidal capillary plexus flow area, [7] others have found a decrease in choroidal capillary plexus flow density in the parafoveal 6 weeks after surgery [8]. The impact of SO on choroidal blood flow is still debated, and it is unclear which layer of the choroidal structure is affected - the vascular layer or the stromal layer [18]. Therefore, an objective and quantifiable parameter is urgently needed to assess the vascular status of the choroid.

To address this need, choroidal vascular index (CVI) was created. By using enhanced depth imaging-optical coherence tomography (EDI-OCT), a clear image of the choroid is obtained, and ImageJ software is used to binarize the image. Black pixels are considered luminal area, while white pixels are considered stromal area. The ratio of luminal area to total choroidal area is called CVI [19, 20]. Unlike CT, CVI is less susceptible to interference and has been used to assess choroidal circulation in several diseases, such as age-related macular degeneration, central serous chorioretinopathy, uveitis, and diabetic retinopathy [12, 19, 21–25]. With increasing evidence, CVI is emerging as a potentially more powerful marker and a complementary tool for assessing choroidal vasculature in various eye diseases. In a retrospective study, CVI was used to evaluate choroidal circulation in RRD patients underwent scleral buckling [26]. In this study, we present the first observation of CVI in complicated RRD patients who underwent vitrectomy and SO tamponade.

## Methods

### Study design and patients

This retrospective study involved patients who received treatment for complicated RRD at The Affiliated Xuzhou Municipal Hospital of Xuzhou Medical University between June 2021 and August 2022. The study was conducted in accordance with the principles of the Declaration of Helsinki, and approval was obtained from the Ethics Committee of the Xuzhou First People’s Hospital. All subjects included in the study provided written informed consent.

The inclusion criteria were as follows: (1) Patients with retinal detachment who had a preoperative PVR classification of **C1** or other situations required vitrectomy combined with SO tamponade, accompanied by successful retinal repositioning. (2) Patients received at least a 2-month follow-up period after SO removal.

Exclusion criteria were as follows: (1) Patients who had undergone previous internal ophthalmic surgery in either of two eyes, except for cataract surgery. (2) Patients with a history of retinal disease, such as choroidal neovascularization, diabetic retinopathy, retinal dystrophy, central serous chorioretinopathy, or glaucoma. (3) Patients with an equivalent spherical lens ≤ − 6 diopters (D) or astigmatism reference ≥1.5 D. (4) Patients who had a duration of more than two months between the chief complaint of RRD and the surgery. (5) Patients with poor image quality or missing medical records.

The following data were recorded from the electronic medical record system: age, sex, preoperative macular status, extent of retinal detachment, duration of patient chief complaints, size of the retinal hole, number of holes, duration of silicone oil tamponade, use of perfluorocarbon liquid, presence or absence of phacoemulsification, logarithm of minimal angle of resolution (LogMAR) BCVA before and after the operation, and postoperative fundus examination. The patients were followed up with a monthly review after the surgery, during which the BCVA, intraocular pressure, and retinal reposition were examined. Notably, patients were required to undergo an EDI-OCT scan twice, once within 1 week prior to SO removal, and the second time during the last follow-up examination.

All SO-filled treated eyes received the same brand of SO Siluron 5000 (FLUORON GmbH, Germany) (density [25 °C]: 0.96 ± 0.02 g/cm^3^, viscosity [25 °C]: 5000–5400 mPa.s, surface tension [35 ± 2 °C]: 21–23 mN/m) by injection.

### Image analysis

**Fig. 1:**
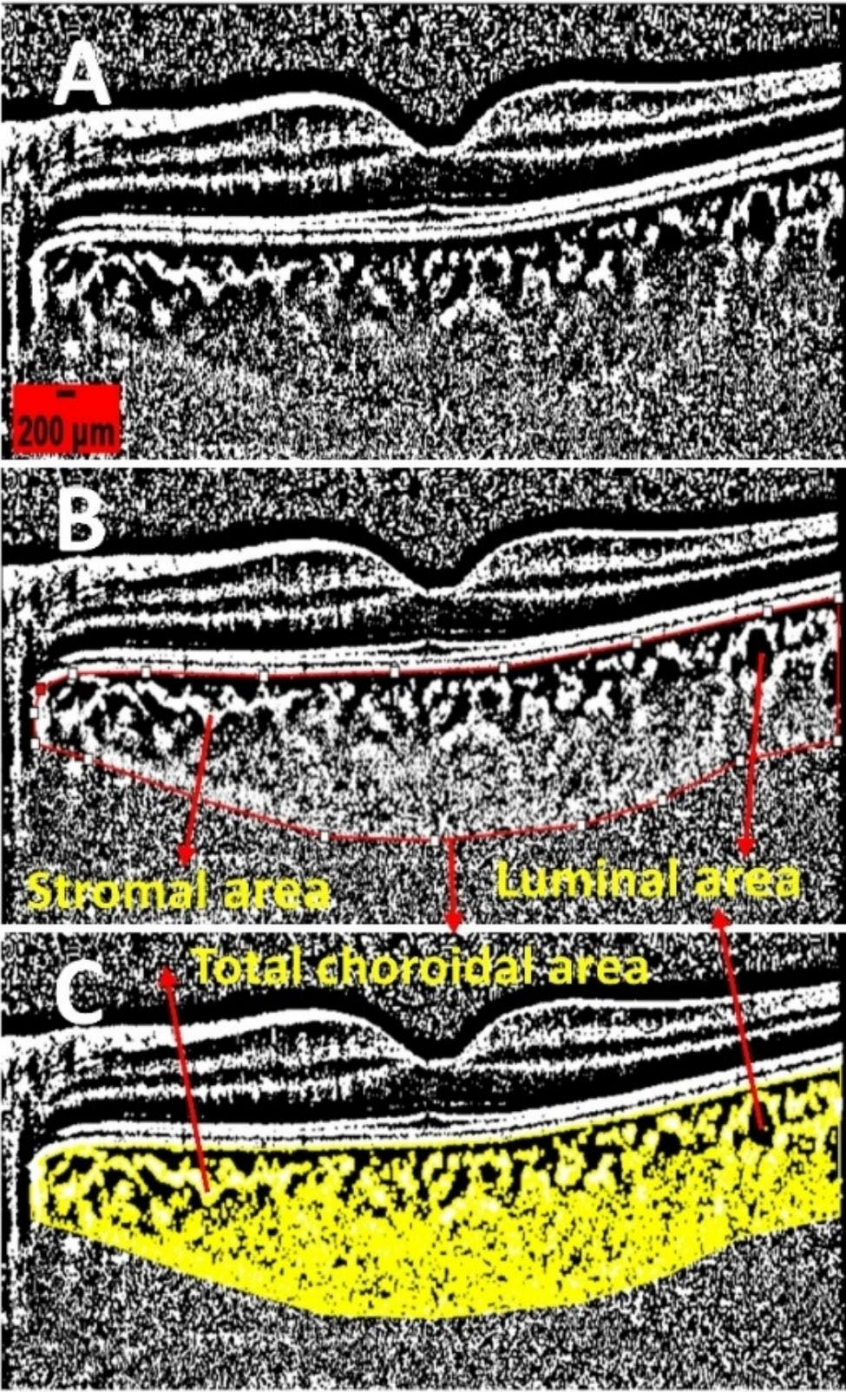
The calculation of CVI using binarization of OCT images. (**A**) The image is binarized using Niblack’s auto local threshold. (**B**) The choroidal boundaries are traced, identifying the total choroidal area (indicated by red lines). (**C**) The color threshold tool is utilized to select the dark pixels, representing the luminal area. The CVI value is obtained by dividing the luminal area by the total choroidal area

All patients underwent EDI-OCT scans using a spectral HRA-OCT device from Heidelberg Engineering, Heidelberg. To avoid diurnal variation in CVI, each session was performed at approximately the same time in the morning. EDI-OCT images were obtained with a 30 × 5° volume scan center on the macular fovea, averaging 100 frames per scan. The EDI-OCT scan passing through the macular fovea was selected for analysis. The choroid was defined as the space between the outer edge of the retinal pigment epithelium and the choroid-scleral junction. After determining the scale using the public domain software Fiji (http://fiji.sc/Fiji), the CT was manually measured by two independent examiners (Meishuang Li and Yumei Cao). With the macular fovea as the center, one position was measured every 750 μm towards the nasal and temporal side, for a total of 5 positions. The average values of the two examiners were taken for statistics [27]. The EDI-OCT images were binarized and segmented by the same examiners using the semi-automated method described previously [25]. In short, the EDI-OCT image was opened in ImageJ, and the correct scale was set. When converting the image to an 8-bit image, the image was binarized using Niblack’s automatic local thresholding (Fig. 1, Part A). The polygon tool was then used to select the region of interest across the length of the EDI-OCT scan (Fig. 1, Part B). The upper boundary of the area of interest was traced along the choroid-retinal pigment epithelial junction, and the lower boundary was traced along the choroid-scleral junction to determine the total choroidal area. The images were converted back to red, green and blue images, and dark pixels representing the luminal area were selected using the color threshold tool (Fig. 1, Part C). Total choroidal area and luminal area were measured, and stromal area was calculated by subtracting luminal area from total choroidal area. CVI was then calculated as (luminal area)/(total choroidal area). Choroidal parameter calculations were performed by two investigators (Meishuang Li and Yumei Cao) separately without knowledge of the patient’s characteristics, and the mean value for each parameter was used for the statistical analysis.

### Statistical analysis

Statistical analysis was performed using SPSS software (version 26.0, IBM SPSS Statistics). Qualitative parameters were described by frequencies and percentages, while quantitative parameters were described by mean and standard deviation or medians and interquartile ranges. The Shapiro-Wilk test was used to test for the normality of distribution. The paired Student’s t-test was used to compare normally distributed continuous variables, whereas the Wilcoxon signed-rank test was used for non-normally distributed variables. The relationship between CVI and duration of SO tamponade was assessed using Pearson correlation analysis and simple linear regression analysis. A p value of < 0.05 was considered statistically significant.

## Results

36 patients were participated in this study, the demographic and clinical characteristics were shown in Table 1. Four patients had macular edema which disappeared after the removal of SO. BCVA was improved after SO removal compared with that noted before SO removal (0.57 ± 0.33 LogMAR vs. 0.72 ± 0.42, P = 0.021).

**Table 1:** Baseline characteristics and intraoperative data of patients. (Data were expressed as mean ± standard deviation)

Characteristic	Value
Age, year	53.3 ± 9.7
Male, n (%)	18(50)
Right Eye, n (%)	24(67)
BCVA (before SO removal), LogMAR	
operated eyes	0.72 ± 0.42
fellow eyes	0.15 ± 0.26 (**P = 0.000**)
IOP (before SO removal), mmHg	
operated eyes	12.97 ± 3.02
fellow eyes	13.13 ± 2.02 (P = 0.761)
Axial length, mm	
operated eyes	24.04 ± 1.53
fellow eyes	24.09 ± 1.36 (P = 0.322)
Duration of RRD before surgery, days	21.19 ± 2.83
Duration of SO tamponade, days	102.19 ± 5.34
Operated eye of BCVA, LogMAR	
Before SO removal	0.72 ± 0.42
After SO removal	0.57 ± 0.33 **(P = 0.021)**
Macular status	
Macular off, n (%)	32(89)
Macular on, n (%)	4(11)
Edema after SO injection, n (%)	4(11)
Use of perfluorocarbon liquid, n (%)	33(92)
Combined cataract extraction, n (%)	
During RRD surgery	21(58)
During SO removal surgery	7(19)
Epiretinal membrane peeling, n (%)	7(19)

**Table 2:** Choroidal parameters of the operated eyes compared with the fellow eyes. (Data were expressed as median [interquartile range] or mean ± standard deviation)

Parameter	Operated eyes	Fellow eyes	P
Luminal area, mm^2^			
Before SO removal	0.909 [0.694; 1.185]	1.091 [0.785; 1.296]	**0.007**
After SO removal	0.994 [0.712; 1.348]	1.034 [0.731; 1.249]	0.683
Total choroidal area, mm^2^			
Before SO removal	1.553 [1.182; 2.029]	1.775 [1.308; 2.148]	0.285
After SO removal	1.629 [1.214; 2.238]	1.678 [1.263; 2.107]	0.975
Stromal area, mm^2^			
Before SO removal	0.612 [0.507; 0.848]	0.684 [0.530; 0.880]	0.730
After SO removal	0.636 [0.495; 0.893]	0.648 [0.521; 0.829]	0.545
CT, µm			
Before SO removal	258.2 [178.2; 323.2]	277.6 [194.3; 344.0]	0.245
After SO removal	243.8 [176.1; 339.0]	265.2 [194.6; 336.4]	0.182
CVI, %			
Before SO removal	57.616 ± 0.030	60.042 ± 0.019	**<0.0001**
After SO removal	59.530 ± 0.018	60.319 ± 0.020	**0.031**

**Table 3:** Choroidal parameters before and after SO removal. (Data were expressed as median [interquartile range] or mean ± standard deviation)

Parameter	Before SO removal	After SO removal	P
Luminal area, mm^2^			
operated eyes	0.909 [0.694; 1.185]	0.994 [0.712; 1.348]	**0.028**
fellow eyes	1.091 [0.785; 1.296]	1.034 [0.731; 1.249]	0.079
Total choroidal area, mm^2^			
operated eyes	1.553 [1.182; 2.029]	1.629 [1.214; 2.238]	0.519
fellow eyes	1.775 [1.308; 2.148]	1.678 [1.263; 2.107]	0.113
Stromal area, mm^2^			
operated eyes	0.612 [0.507; 0.848]	0.636 [0.495; 0.893]	0.338
fellow eyes	0.684 [0.530; 0.880]	0.648 [0.521; 0.829]	0.068
CT, µm			
operated eyes	258.2 [178.2; 323.2]	243.8 [176.1; 339.0]	0.875
fellow eyes	277.6 [194.3; 344.0]	265.2 [194.6; 336.4]	0.227
CVI, %			
operated eyes	57.616 ± 0.030	59.530 ± 0.018	**0.0003**
fellow eyes	60.042 ± 0.019	60.319 ± 0.020	0.379

The study analyzed the choroidal parameters of both operated eyes and fellow eyes before and after SO removal, with the data presented in Tables 2 and 3. These tables represent two distinct methods for analyzing the choroidal parameters.

Table 2 showed that the luminal area of operated eyes before SO removal was lower than that of fellow eyes (0.909 [0.694; 1.185] vs. 1.091[0.785; 1.296], P = 0.007). However, the difference between the two after SO removal was not statistically significant (P = 0.683). In contrast, Table 3 demonstrated that the luminal area before SO removal was lower in operated eyes than after SO removal (0.909 [0.694; 1.185] vs. 0.994[0.712; 1.348], P = 0.028). The luminal area before and after SO removal was not different in the fellow eyes (P = 0.079).

Regarding the total choroidal area and stromal area, Table 2 revealed no statistically significant differences between operated eyes and fellow eyes before SO removal (P = 0.285 and P = 0.730). Similarly, the difference between operated eyes and fellow eyes after SO removal was not statistically significant for total choroidal area and stromal area (P = 0.975 and P = 0.545). In Table 3, the total choroidal area and stromal area for operated eyes before SO removal compared to after SO removal were not statistically significant (P = 0.519 and P = 0.338, respectively). The same was true for fellow eyes (P = 0.113 and P = 0.068, respectively).

For the analysis of CT, Table 2 showed that both before and after SO removal, the CT of operated eyes, although lower than that of fellow eyes, was not statistically significant (P = 0.245; P = 0.182). As shown in Table 3, the CT of operated eyes before and after SO removal was not statistically significant (P = 0.875). The same result was found for the CT of fellow eyes (P = 0.227). No significant differences in CT were noted between the groups.

The analysis of CVI showed that in Table 2, the CVI of the operated eyes was significantly lower than that of fellow eyes before SO removal (57.616 ± 0.030% vs. 60.042 ± 0.019%, P < 0.0001) and remained lower in operated eyes than in fellow eyes after SO removal (59.530 ± 0.018% vs. 60.319 ± 0.020%, P = 0.031). In Table 3, the CVI of operated eyes before SO removal was also lower than that after SO removal (57.616 ± 0.030% vs. 59.530 ± 0.018%, P = 0.0003). The CVI of fellow eyes before and after SO removal was not statistically significant (P = 0.379).

**Fig. 2:**
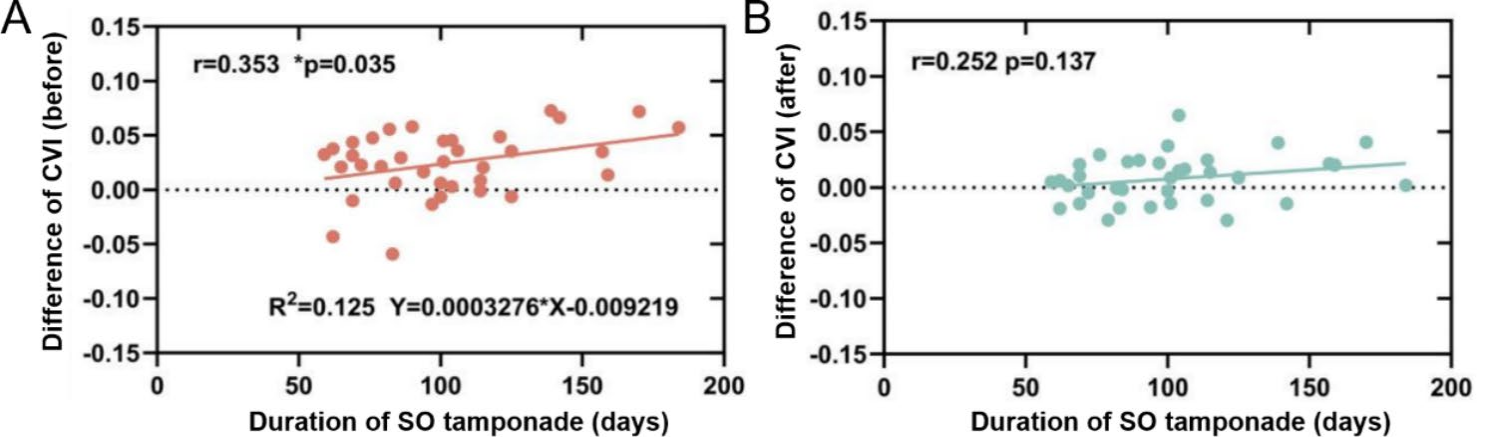
Duration of SO tamponade *versus* CVI difference of the fellow eyes and operated eyes (**A**) before and (**B**) after SO removal

**Fig. 3:**
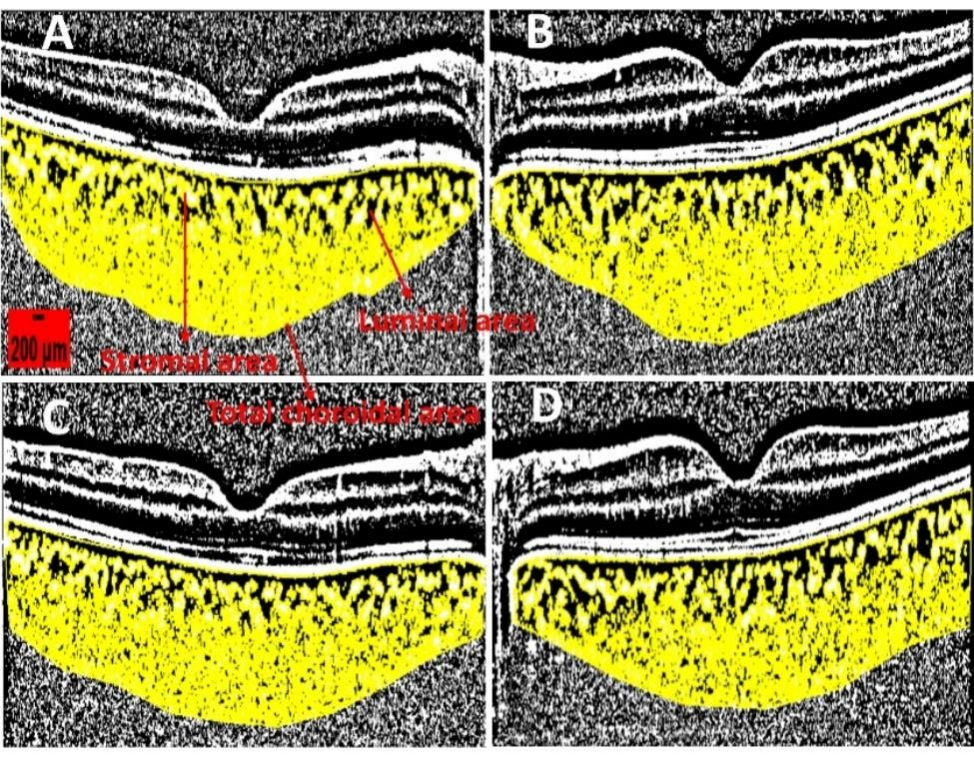
A representative case of a 31-year-old female patient with macula-off RRD in her right eye who underwent vitrectomy combined with SO tamponade. The duration of SO tamponade was 76 days. The BCVA before SO removal was 0.5 LogMAR (operated eye) and 0 LogMAR (fellow eye). After SO removal, the BCVA was 0.4 LogMAR (operated eye) and 0 LogMAR (fellow eye). The IOP before SO removal was 10 mmHg (operated eye) and 13 mmHg (fellow eye), while after SO removal it was 11 mmHg (operated eye) and 12 mmHg (fellow eye). The calculation of CVI using binarization of OCT images is shown. The CVI is obtained by dividing the luminal area by the total choroidal area. (**A**) Displays the CVI of the operated eye before SO removal, with a CVI (%) value of 58.416 (1.26467/2.16496). (**B**) Shows the CVI of the fellow eye at the same time point as A, with a CVI (%) value of 63.171 (1.38585/2.19383), which is higher than A. (**C**) Demonstrates the CVI of the operated eye after SO removal, with a CVI (%) value of 59.590 (1.36166/2.28504), which is higher than A. **D**) Exhibits the CVI of the fellow eye at the same time point as C, with a CVI (%) value of 62.516 (1.35600/2.16904), which is higher than C

Pearson correlation analysis showed that SO tamponade time and the CVI difference of fellow eyes and operated eyes were positively correlated before SO removal (r = 0.353, P = 0.035), with a simple linear relationship between the two (R^2^ = 0.125, Y = 0.0003276*X-0.009219). However, no correlation was noted between SO tamponade time and the CVI difference between fellow eyes and operated eyes after SO removal (r = 0.252, P = 0.137). The results are presented in Fig. 2A and B. A typical example is showed in Fig. 3.

## Discussion

In the past, most scholars used CT to study the choroid. In this study, we applied a new method that allows for the measurement of the vascular and stromal area of the choroid [25]. We found that in the SO-filled state, the CVI was significantly lower in operated eyes. After SO removal, although the CVI increased, it was still statistically lower than in fellow eyes. We found that the change in the CVI was mainly influenced by changes in the luminal area, and no significant differences in the total choroidal area and stromal area were noted in operated eyes and fellow eyes before and after SO removal. It is reasonable to believe that SO mainly affects the luminal area in the choroid and thus causes changes in the CVI. However, after the removal of SO, there was no statistical difference in luminal area between operated eyes and fellow eyes, whereas there was a statistical difference for CVI. By comparing the difference of each parameter with the fellow eyes after SO removal, we found that the difference in luminal area was the largest compared to the stromal area. This finding provides further evidence that SO mainly affects the change in luminal area and thus the CVI.

A retrospective cross-sectional study found that patients with RRD receiving SB treatment exhibited an increased total choroidal area, luminal area, and stromal area compared to the fellow eyes, whereas no differences in the CVI were noted [26]. This study focused on the same disease as our study. However, the results are different due to the use of different treatment methods, which further confirms that the reduction in the CVI and luminal area observed in our study is mainly due to SO tamponade. Based on our findings, the choroidal vascularity index (CVI) of the operated eyes significantly increased after silicone oil (SO) removal compared to before removal (P = 0.0003, Table 3). This indicates that there is an effect of SO on the CVI of the operated eye. Additionally, the CVI of the operated eyes was significantly lower than the CVI of the fellow eyes before SO removal (P<0.0001, Table 2). However, even after SO removal, the CVI of the operated eyes remained significantly lower than that of the fellow eyes (P = 0.031, Table 2). In our opinion, the difference in CVI between the operated eyes and the fellow eyes after SO removal can be mainly attributed to influential factors such as rhegmatogenous retinal detachment (RD) and surgical trauma. The presence of SO tamponade accentuates the difference in CVI between the operated eyes and the fellow eyes (P<0.0001 vs. P = 0.031, Table 2), making it more significant.

We also found that the difference between the CVI of operated eyes and fellow eyes during SO tamponade increased as the duration of SO tamponade increased. The difference between the CVI of operated eyes and fellow eyes after SO removal did not correlate with the duration of SO tamponade. Previously, a positive correlation between SO tamponade duration and the area of the retina FAZ was reported, which suggested that an increase in SO tamponade duration led to an increase in the area of the FAZ [6]. As the choroid is the source of blood supply to the FAZ, the relationship between CVI and the area of the FAZ needs to be considered. Previously, a study showed that CVI was negatively associated with the area of the FAZ in healthy subjects [28]. We believe that changes in CVI after SO tamponade in complicated RRD eyes affect changes in the area of the FAZ of the retina, resulting in poor visual prognosis. For example, unexplained vision loss after SO tamponade or removal has recently been reported in eyes, and the duration of SO tamponade was considered to be the only risk factor [29, 30]. Microvisual field abnormalities and macular central dark spots have been found in such patients. We speculate that this phenomenon may be related to changes in CVI. Previously, there was no clear explanation for the unexplained vision loss caused by SO tamponade. Initially, it was thought that the mechanical stress of SO caused ischaemic apoptosis in retinal cells [31]. Second, it has been suggested that SO can infiltrate tissue, leading to optic nerve damage and macular dysfunction [32, 33]. In addition, emulsified SO can break the inner limiting membrane and enter the retina, thinning the retina and even infiltrating the optic nerve and migrating into the brain [34, 35]. It is also believed that SO replaces the vitreous humour but does not buffer changes in ion levels within the vitreous cavity. For example, the failure of Müller cells to siphon potassium ions leads to potassium ion accumulation [36]. It has even been suggested that the hydrophobicity of SO causes retinal dehydration [5]. SO transmits more blue light, leading to phototoxicity, [37, 38] and SO stimulates the production of a large number of phagocytes and pigment cells, leading to an increase in inflammatory mediators that cause retinal cell denaturation and necrosis [39]. However, there is no clear and uniform answer to this question. Our study provides an alternative interpretation to this phenomenon and suggests that SO potentially affects choroidal blood flow, leading to an increase in the area of the FAZ and thus causing dark spots in the macula and microvisual field abnormalities in patients with unexplained vision loss.

In the past, CT was considered an indicator of choroidal structure. In our study, there was no statistical difference between CT and fellow eyes before and after removal of SO, as the duration of SO tamponade did not last more than 6 months (102.19 ± 5.34 days). Previous studies have revealed CT differences between operated eyes and fellow eyes or before and after SO removal, which were considered statistically different after 6 months of SO tamponade [13, 15]. This is consistent with the findings of our study. It is seen here that CVI is more sensitive than CT. In addition, CT is influenced by many factors and cannot identify the choroidal vessels and stroma [12]. CVI can give better insight into choroidal blood circulation.

The retrospective design is the limitation of this study. We are now implementing a randomized controlled trial (ClinicalTrials.gov identifier CHiCTR2200059077) to investigate the effect of SO tamponade duration on CVI. In the future, we will focus on the mechanisms of this effect and conduct experimental studies.

## Conclusions

In conclusion, the data could suggest that the SO tamponade reduces CVI and decreases choroidal blood circulation in patients with retinal detachments requiring vitrectomy combined with SO tamponade. The longer the SO tamponade time, the more CVI reduction.

## Data Availability

The datasets generated during and/or analyzed during the current study are available from the corresponding author on reasonable request.
